# Learning and engagement through natural history museums^*^

**DOI:** 10.1080/03057267.2018.1442820

**Published:** 2018-03-15

**Authors:** Tamjid Mujtaba, Martin Lawrence, Mary Oliver, Michael J. Reiss

**Affiliations:** a UCL Institute of Education, University College London, London, UK; b Natural History Museum, London, UK; c School of Education, University of Nottingham, Nottingham, UK

**Keywords:** Natural history museums, informal science, digital technologies, learning, engagement

## Abstract

This review examines how natural history museums (NHMs) can enhance learning and engagement in science, particularly for school-age students. First, we describe the learning potential of informal science learning institutions in general, then we focus on NHMs. We review the possible benefits of interactions between schools and NHMs, and the potential for NHMs to teach about challenging issues such as evolution and climate change and to use digital technologies to augment more traditional artefacts. We conclude that NHMs can provide students with new knowledge and perspectives, with impacts that can last for years. Through visits and their on-line presence, NHMs can help students see science in ways that the school classroom rarely can, with opportunities to meet scientists, explore whole topic exhibitions, engage with interactive displays and employ digital technologies both *in situ* and to support learning in the school science classroom. Although these interactions have the potential to foster positive cognitive, affective and social outcomes for students, there is a lack of reliable measures of the impact of NHM experiences for students. Opportunities to foster relationships between NHM staff and teachers through professional development can help articulate shared goals to support students’ learning and engagement.

## Introduction

The purpose of this review is to identify key factors that impact the learning potential of natural history museums (NHMs), with a particular focus on school audiences (K-12, from ages approximately 5 to 17 years). To place NHMs in the broader informal environment, we first provide an overview of the research on informal science learning (ISL) and discuss models of relationships between formal and ISL institutions. We then consider the role of NHMs with respect to cognitive, affective and social aspects of learning, and detail the scope of recent NHM exhibitions. We go on to highlight the role NHMs can play in the professional development of teachers. Finally, we consider ways to support learning experiences of visitors to NHMs. We concentrate on what may be termed ‘student learning’ – that is, learning by those of school age. However, some of this learning does not take place while students are on school visits to NHMs or in schools being taught by experts from NHMs. Rather, it occurs as students visit or otherwise access NHM resources individually, with their families or with friends. Furthermore, it is increasingly the case that some such learning takes place remotely through new digital technologies. Throughout, we present findings from the general literature about learning in and through NHMs, as well as other ISL institutions when these have implications for the learning that takes place through NHMs.

The literature search drew from a variety of sources, including ‘grey’ literature, using the following search terms: learning in natural history museums; learning in science museums; learning in aquaria; learning in informal science institutions; exhibits natural history museums; programmes natural history museums; education natural history museums/informal science institutions; students and natural history museums/informal science institutions and schools and natural history museums/informal science institutions. Electronic databases included Oxford Journals Collection, SpringerLink, JSTOR, Cambridge Journals Online, Sage Journals and Taylor and Francis Online and with an internet search engine to access unpublished material, including that produced by NHMs themselves. The many terms are associated with ‘learning out of school’. We use ISL as a general term but have a particular focus in this paper on understanding the role of NHMs as places of learning for school-aged students. As research institutions and public-facing entities of science, NHMs are at the forefront of science and possess insights, narratives, materials and knowledge that can benefit science learning. Traditionally seen as sites of ISL, NHMs increasingly embody formal approaches, to the extent that some even run higher education courses and award degrees.

Research in the UK, US and other countries has found mounting evidence that development of students’ knowledge and understanding of scientific concepts takes place in a variety of settings – both in and out of school – and that such knowledge and understanding accumulate over time through exposure to a wide range of public resources, from museums to the media (e.g. Barron, 2006; Bathgate, Schunn, & Correnti, 2014; Bell, Lewenstein, Shouse, & Feder, 2009; Downey, Von Hippel, & Broh, 2004; Falk & Dierking, 2010; Falk & Needham, 2013; Organization for Economic Co-operation and Development [OECD], 2012; Tal & Dierking, 2014). One view of ISL is that

Informal science learning refers to activities that occur outside the school setting, are not developed primarily for school use, are not developed to be part of an ongoing school curriculum, and are characterised as voluntary as opposed to mandatory participation as part of a credited school experience. (Crane, Nicholson, Chen, & Bitgood, 1994, p. 3)

We include in this review learning experiences that are offered outside formal school settings. Experiences with ISL can, for some, represent their primary exposure to science and their first real experience of science learning (Bell et al., 2009), and such experiences can play a key role in the development of skills, dispositions, practices and knowledge in helping students to learn about science (Dorph, Schunn, Crowley, & Shields, 2012). Clear evidence for this comes, for example, from the US National Research Council report *Learning Science in Informal Environments* (Bell et al., 2009). This report indicates that community institutions (such as museums) that support science learning are able to support young people’s learning of and interest in science better than schools working in isolation from such organisations. Braund and Reiss (2006) recommended that students should be presented with opportunities for engaging with ISL environments and have opportunities to visit zoos, science centres, museums and botanic gardens. Data from international tests such as the Programme for International Student Assessment (PISA) show that ISL experiences are positively associated with interest and achievement in science (McConney, Oliver, Woods-McConney, Schibeci, & Maor, 2014). Rather than being an ‘add-on’ to formal learning, ISL can enhance and enrich student learning. So how might learning in formal and informal settings be characterised? What makes them different or distinct?

## The role of ISL for learners and teachers

Attempts to examine the similarities and differences between learning in formal and informal contexts have focused on context, affective and social measures, the nature of participation and curriculum content (Martin, 2004; Stocklmayer, Rennie, & Gilbert, 2010; Wellington, 1990). There has been a recent emphasis on the trend to integrate or blend learning experiences for students through the use of closer working between host ISL and school institutions, and through the use of digital technologies. ISL institutions such as museums have developed a range of methods to help with formal science learning. A large number of ISL institutions, for example, offer science education programmes, often in the form of field trips that are available to schools and can bridge the learning that takes place in informal and formal settings. An Informal Science Education ad hoc Committee of the Board of the National Association for Research in Science Teaching (NARST) issued a statement indicating that:

Learning rarely if ever occurs and develops from a single experience. Rather, learning in general, and science learning in particular, is cumulative, emerging over time through myriad human experiences, including but not limited to experiences in museums and schools; while watching television, reading newspapers and books, conversing with friends and family; and increasingly frequently, through interactions with the Internet. The experiences children and adults have in these various situations dynamically interact to influence the ways individuals construct scientific knowledge, attitudes, behaviors, and understanding. In this view, learning is an organic, dynamic, never-ending, and holistic phenomenon of constructing personal meaning. This broad view of learning recognizes that much of what people come to know about the world, including the world of science content and process, derives from real-world experiences within a diversity of appropriate physical and social contexts, motivated by an intrinsic desire to learn. (Dierking, Falk, Rennie, Anderson, & Ellenbogen, 2003, p. 109)

Continued efforts in bridging informal and formal learning environments to increase students’ engagement and interest in science have been proposed by the European European Commission (2007). With active research programmes, NHMs in particular are committed to and geared towards helping students engage with real world science with opportunities for students to know about the societal and scientific challenges of the present and future world.

Lawrence and Tinkler (2015), using the Natural History Museum in London as an example, demonstrated that science museums are no longer representing a static image of science or a fixed body of knowledge. Changes and developments in science museums have led to opportunities for students to gain more knowledge about the various processes of doing science – to engage directly with scientists and real scientific activities. NHMs are ideally placed to help students learn more about science and to educate the public about important issues such as biodiversity, an area that requires special attention given the lack of public awareness about how such issues impact countries (Natural History Museum, London, 2015). For example, to care more about biodiversity loss, the public needs to be emotionally and intellectually engaged, informed about threats and aware of possible remedial actions (Natural History Museum, London, 2015). Whilst the remit and scope of NHMs extend beyond addressing the needs of school students, it is the interaction between these institutions and schools that is the primary focus here.

## Interactions between schools and NHMs

The literature on interactions between schools and NHMs is modest in extent. However, that on interactions between schools and museums of any type is larger and, in the face of evidence to the contrary, it seems a priori likely that conclusions reached with respect to museums in general also apply to NHMs.

Research exploring interactions between museum educators, students and teachers indicates that often a school group is led by a museum educator, who in turn provides content but encourages little social interaction between the various members of the group (Cox-Petersen, Marsh, Kisiel, & Melber, 2003). Even when a teacher is leading the visit by using task sheets, social interactions between teachers, students and chaperones are not common (Griffin & Symington, 1997; Kisiel, 2003). In the US, a number of organisations (e.g. Institute of Museum and Library Services, 2005) have strongly recommended that ISL institutions (such as museums) are used to support students’ learning of science, with increasing recognition that formal partnerships between schools and informal settings is an effective way to help students with their science education (Bobick & Hornby, 2013; Pumpian, Fisher, & Wachowiak, 2006). A report by the Centre for the Advancement of Informal Science Education (Bevan et al., 2010), which closely examined US partnerships between schools and informal science education settings, indicated that despite the occurrence of partnerships between schools and ISL institutions there was little evidence to show the impact or sustainability of such partnerships.

Falk, Needham, Dierking, and Prendergast (2014) conducted an extensive review to explore how connected the science education community is in the UK. Their findings indicate that certain parts of the science education community, notably those in the informal sector, are highly to moderately interconnected and collaborative, but that this is not the case with universities and schools. Whilst schools benefit from ISL institutions, and all sectors contribute to the needs of schools, schools themselves return very little back and very little partnering between schools occurs. In contrast, ISL institutions such as museums were well interconnected and worked collaboratively. Falk and colleagues concluded that, in order to maximise the effectiveness of science education, those involved within various communities would need to develop and build collaborative and synergistic relationships. They also found that there is much effort placed on developing resources for school-aged students but very little for those under the age of five and for adults. Other inequalities included fewer resources developed for those in rural communities and those from lower socioeconomic backgrounds. Where partnerships are being or have been established, in order to engage schools and their students fully, NHMs needed to take account of individual school culture (Anderson, Kisiel, & Storksdieck, 2006; DeWitt & Osborne, 2007; Mortensen & Smart, 2007; Tal & Morag, 2007).

Partnerships and interactions between schools and museums present many challenges, as has been well documented (Anderson et al., 2006; Davidson, Passmore, & Anderson, 2010; DeWitt & Storksdieck, 2008; Griffin & Symington, 1997; Kang, Anderson, & Wu, 2009; Phillips, Finkelstein, & Wever-Frerichs, 2007; Tal, Bamberger, & Morag, 2005). For example, in an observational and interview study of 40 class visits to four NHMs in Israel, researchers reported that most teachers were unable to indicate why they had chosen to take students on a field trip to the museum, as they had neither planned the trip nor chosen the learning activities (Tal et al., 2005). They also found that some teachers did not view the field trip as a well-planned educational experience but rather as a ‘fun’ event. In this study, Tal et al. (2005) reported that a very few teachers had actually spent time on planning the trip and relating it to the class curriculum, nor had they planned the trip because it linked with what they were teaching in class. Pre-visit activities for their students were also not created.

Research has clarified the roles of schools and museums in creating successful partnerships from both teachers’ and museums’ perspectives (DeWitt & Osborne, 2007; Gupta, Adams, Kisiel, & Dewitt, 2010; Kisiel, 2010; Tal & Steiner, 2006; Tran, 2007). What teachers believe what would be useful for their students’ learning goals are often not fully realised in field trips to informal institutions due to a misalignment in the expectations of school teachers and museum staff. For example, Kisiel (2005) found that whilst 90% of teachers who took their students on a trip to an informal education institution indicated that they had hoped the trip would complement the school’s curriculum goals, only 23% of them found that this was the case. Tal and Steiner (2006) conducted observations and interviews of teachers on a field trip at a science museum, noting that the teachers had not taken a lead or active role in directing students’ learning, leaving this to the museum educators. In turn, the museum educators had expected teachers to be more active in their students’ learning. Interviews and observations were used by Tran (2007), who explored interactions between teachers and museum staff in order to learn more about the practices and perspectives of the museum staff. The findings indicated that the two key aims of the museum educators’ efforts were to encourage students to return to the museum whilst also helping to foster and develop students’ interest in science. For museum educators, it was more important to provide a meaningful and memorable experience than developing students’ science content knowledge. In this particular study, there were differentiated roles for the museum educators and teachers, with museum educators expected to take the lead in educating students and teachers maintaining responsibility for managing student behaviour and time. Similar results have been reported by Weiland and Akerson (2013), reporting on a collaboration and development of an elementary classroom science unit, where good communication between teachers and museum educators existed and respective roles had been established prior to the implementation of the unit. Likewise, Kisiel (2010) reported on a partnership where a school was given access to an aquarium for field trips alongside aquarium-led science lessons for two years.

Establishing effective partnerships takes good communication; it generally requires changes in organisational practices where differences in institutional practice can be obstacles to more effective relationships (Kisiel, 2010). Such differences arise because teachers and museum educators work in very different contexts, perhaps without fully understanding the particular constraints and challenges within which each work (DeWitt & Osborne, 2007). DeWitt and Osborne suggested a Framework for Museum Practice (FMP), which drew attention to the need for the resources in museums to be in alignment with the requirements of teachers (for curriculum needs), whilst also making full use of educational resources found in museums. As well as maximising the use of museum resources to support student learning, clarification about the purpose of a visit to an NHM is needed.

Kisiel (2014) found that different categories of members of informal science education organisations varied in their ideas of what successful activities would involve. Whilst volunteers at a museum may equate a successful visit with good student behaviour, informal science educators are more likely to suggest that a successful activity will introduce particular standards-based concepts (e.g. evolution). These expectations were found to differ from those of teachers, who viewed a successful visit as providing students with exposure to a new learning environment. Kisiel indicated that another barrier to a successful partnership is that both teachers and informal science educators may not be sure about which activities are ‘possible’. For partnerships to be successful, teachers need to ‘cross boundaries’ (Aikenhead, 2006) into multiple communities in order to be able to incorporate resources from ISL organisations into their practice. In the same way, ISL educators need to navigate through school communities as well as their own organisation in order to foster effective connections with schools and/or teachers. This way of working, suggested by Wenger (1998), indicates that for one community (e.g. museums) to interact successfully with another community relies on both communities clarifying boundaries, as well as defining strategies for encounters or crossing those boundaries.

## Evaluation of the experience: affective, cognitive or social?

NHMs routinely collect evaluation data from visitors, whether to improve the ‘visitor experience’, to gauge interest or research the learning that has taken place (Natural History Museum, London, 2015). Measuring learning outcomes at ISL institutions and determining the effectiveness of the learning experience is important but, to date, defining a good learning outcome has often proved contentious (Bell et al., 2009). Bell and colleagues pointed out that achievement measures used in school settings have been used as tools to measure outcomes at ISL institutions but argue that it is not appropriate to use traditional academic achievement outcomes because they do not reflect the defining characteristics of informal environments. Informal settings potentially provide a range of outcomes that are not possible to measure with typical academic achievement instruments. The curriculum of informal settings differs markedly from that of schools and as the learning is often voluntary, this means that academic measures are inappropriate markers of these unique experiences. Finally, exhibits are designed for a range of ages and abilities, and for specific learning and experiential goals within their defined spaces. Capturing this experience with academic outcomes is problematic. Bell and colleagues suggested that rather than using academic outcomes or subjective learning goals, educators and researchers should develop a variety of specialised science learning goals based on a framework developed in the US for K-8 science learning (National Research Council, 2007). Bell and colleagues identify six strands that capture an effective ISL experience:

Strand 1: Experience excitement, interest, and motivation to learn about phenomena in the natural and physical world (this strand is of particular relevance to informal environments).

Strand 2: Come to generate, understand, remember, and use concepts, explanations, arguments, models, and facts related to science.

Strand 3: Manipulate, test, explore, predict, question, observe, and make sense of the natural and physical world.

Strand 4: Reflect on science as a way of knowing; on processes, concepts and institutions of science; and on their own process of learning about phenomena.

Strand 5: Participate in scientific activities and learning practices with others, using scientific language and tools.

Strand 6: Think about themselves as science learners and develop an identity as someone who knows about, uses, and sometimes contributes to science (this strand is of particular relevance to informal environments). (Bell et al., 2009, p. 4)

In the ISL community (and also the broader science education community) there is a range of views about *how* to measure outcomes appropriately. Although there is a lack of consensus as to which outcomes are the most important ones to measure, there is some agreement among science educators about the *nature* of ISL outcomes. To date there is a shortage of valid, reliable and comprehensive measures that capture learning in NHMs. In this, we recognise the complexity of the outcomes, the diversity of learners and the desire to capture both the explicit and perhaps the ‘hidden’ aspects of the learning experience. Often, learning outcomes are based on the goals and objectives of a programme set by a teacher or informal science educator. However, as is the case in ISL settings, learning is to a large extent inevitably guided by students’ interests and what they choose to focus on during an NHM field trip; this may or may not be closely related to the planned learning objectives. Accordingly, when measuring learning outcomes, researchers should focus on the different kinds of learning that may have taken place during a field activity.

An important point about measuring outcomes is that the long-term impact is not immediately evident. The great majority of studies focus only on short-term outcomes. In addition, Bell et al. (2009) propose that assessments of learning in informal science education environments should measure a range of outcomes including cognitive, behavioural, social, intellectual, attitudinal and participatory capabilities. They also suggest that assessments should include learning experiences that are engaging, as well as having construct and ecological validity. To date, much of the research suggests that there is an over-emphasis on academic outcomes, which may not be ecologically valid for the type of learning that has taken place. Assessments should ideally be aligned with the type of learning that has taken place. In order to make accurate inferences about what has been learnt, and before conclusions can be drawn about the effectiveness of learning in an informal science education environment, the learning activities and the assessment need to be well-aligned. Furthermore, in view of the relationship between engagement and learning, researchers should consider whether the assessments are based on the same norms as those that promote engagement in the informal activities.

## ‘Hooks’ and ‘wonder’

In this section, we address issues to do with engagement, noting that a common claim for museums in general and NHMs in particular is that they are particularly capable of engaging visitors. Whilst NHMs have traditionally showcased physical artefacts, the application of digital technologies in museum settings, including NHMs, is becoming more common and is considered here. A related possibility is that incorporating game-based technologies may make it easier for students to discover that science is meaningful and relevant to their daily lives (Feinstein, Allen, & Jenkins, 2013).

A certain amount of research has been conducted about the particular factors that contribute to successful engagement around exhibits, whether for visitors in general or students specifically. Such research poses methodological challenges as groups dynamically form, overlap, transform and disperse as the result of a naturalistic flow of visitors. In one study, quantitative evidence was gathered in order to identify factors contributing to visitor engagement and learning around interactive surfaces (Block et al., 2015). Block and his colleagues categorised visitors through an automatic grouping algorithm that partitioned a constant flow of visitors into groups based on visitors’ shared time spent around a multitouch exhibit table. The algorithm was developed based on observations of visitor engagement over a two-year period at two exhibits. Age of visitors and occurrence of certain social behaviours, such as turn taking and group size, impacted how visitors engaged with the exhibits (Block et al., 2015). Interestingly, groups of two visitors spent longer on the exhibits than those who were alone or in groups of three. With regards to children, Block et al. (2015) found they were more likely to engage with the scientific content of the exhibits and to spend more time there when adults accompanied them. To maximise effectiveness of these adult-children groups, the authors recommended that information be placed alongside exhibit displays so that adults can help as facilitators to guide children.

Several research studies indicate that visitors to informal environments, including NHMs, greatly appreciate both the entertainment and social aspects of their visits alongside the opportunity for learning. Intrinsic reasons (such as enjoyment) are one of the key reasons why people visit informal environments. The challenges incorporated into interactive exhibits can elicit feelings of positive engagement (Sadler, 2006) and even after the visit has ended people may continue to express interest and excitement about what they have learnt (Stocklmayer & Gilbert, 2002), particularly when they have increased their skills and knowledge (Falk, Scott, Dierking, Rennie, & Jones, 2004).

The use of narrative has been found to be a key element in increasing engagement with scientific concepts in zoos and NHMs (Tunnicliffe, 1996; Tunnicliffe, Lucas, & Osborne, 1997). In a series of studies, Tunnicliffe documented visitors’ use of narrative as a way of gaining knowledge, raising interest and increasing engagement with others when discussing specimens at NHMs and zoos. The intrinsic reasons for visiting informal environments, alongside the intellectual merits of engaging in such activities, have been summarised by Bell et al. (2009):

There is evidence of learner excitement and strong positive emotional responses … There is also clear evidence for learning science content … participants can reflect on the enterprise of science and on their own thinking about science … there is evidence of learners’ attempts to personalise and integrate science learning experiences with their values and identity. (pp. 161, 162)

Given that NHMs invest significant resources in attempting to engage school students, the affective value of visits to them have received surprisingly limited attention. The term ‘affective learning’ has been defined as meaning both the changes in visitors’ attitudes and the emotions that are created by the learning that takes place at ISL institutions (Roberts, 1993). Affective measures (interest, enjoyment, motivation and career aspirations) have been reported to be positively impacted by interactions with museum or science centre exhibits. When Heureka, a Finnish science centre, created opportunities for students to engage in the open-learning environments of the centre, students’ intrinsic motivation was increased (Salmi, 2003). A survey of 1019 undergraduate students at the University of Helsinki showed that informal science education institutions had a strong impact on the academic career choices of students (Salmi, 2003). This finding mirrors the PISA reports, referred to above, of association between ISL experiences and interest in science as a career (McConney et al., 2014).

In a series of 75 interviews with museum professionals, Spock (2000) reported that museum learning experiences (some of which took place in childhood) were life-changing incidents for many of these professionals. Even young children expressed positive affective outcomes after visiting a zoo (Birney, 1988). Hooper-Greenhill et al. (2006) found that both students and teachers indicated positive emotional responses following their participation in museum visits. Winterbotham (2005) conducted a survey of 450 teachers that explored teachers’ perceptions of what they expected their students to gain from museum visits. The teachers expected their students to develop skills and positive attitudes towards the particular subject they wanted to study, particularly when they had had the opportunity to take part in interactive exhibits and handle museum artefacts (Hooper-Greenhill et al., 2006).

The educational value of trips to museums and afterschool programmes at museums has been reported as being reflected in an increase in students’ performance in school science as well the development of more positive attitudes towards science careers, and an increase in interest and self-confidence (Braund, 2004a, 2004b; Murray & Reiss, 2005; Woods-McConney, Oliver, McConney, Maor, & Schibeci, 2011). For example, the Miami Science Museum’s youth programme focuses on young people from low socio-economic backgrounds and gives them the opportunity to improve their interpersonal and communication skills while also providing them with work experience, mentoring and training. Several case studies have demonstrated that the approach used by the Miami Science Museum has a positive impact on students’ grades at the end of compulsory education as well as enhancing their employment opportunities. Similarly, the *Evolutions Afterschool Programme* at the Yale Peabody Museum of Natural History has shown consistently positive impacts on its participants and their interest in and attitudes toward science. Altmann, Tamez, and Bartels (2001) argue that students’ increased school success and enhanced intrinsic motivation in the San Francisco Exploratorium’s programmes is related to the programme’s goals of fostering students’ autonomy and providing an atmosphere of responsibility and respect.

When museums and other informal organisations work with schools using particular interventions during school time there can be long-term benefits for students. For example, Laursen, Liston, Thiry, and Graf (2007) found that even after a short science intervention, teachers reported that students’ engagement and interest and scientific and critical thinking skills had increased for both ‘high-’ and ‘low-’ ability students. In addition, the intervention was able to change students’ stereotypical perceptions of science and scientists. The intervention also had a positive impact on teachers’ increased understanding of new ways to teach science in the classroom. A number of earlier review articles have suggested that museums have not always provided optimal learning experiences (e.g. Bitgood, Serrell, & Thompson, 1994; Falk & Dierking, 1992; Ramey-Gassert, Walberg, & Walberg, 1994). One feature of these studies is that assessment of students’ museum learning used short-term measures only, and focused on the recall of facts and concepts. It may well that the most meaningful learning that takes place at museums, including NHMs, only becomes apparent at a later date, which can stem from anywhere between weeks to a few years after one or more visits (Dierking, Falk, & Abrams, 1996; Falk & Holland, 1994; Falk, Luke, & Abrams, 1996; McManus, 1993).

Interactive exhibits found in NHMs can develop students’ skills as well as create an interest in science in ways that complement learning in school, particularly given that the knowledge and skills gained from the informal science education sector cannot be easily replicated within the classroom. In the *Student Review of the Science Curriculum*, Murray and Reiss (2005) found that museums had a positive impact on 16 to 19 year-old students’ attitudes to science. This review also found that young learners reported that science videos and taking part in hands-on activities were enjoyable. When asked which were the most useful and effective in helping them understand school science, these students reported having a discussion/debate in class, taking notes from the teacher, and doing a science experiment in class. Collins and Lee (2006) maintain that these findings have important implications for museums, including NHMs, suggesting that museums could use discussions and debate as a way of engaging students with learning on museum visits. NHMs often have good links with the scientific community, and these could be used to help set up exhibits that are able to address these issues and, using interactive exhibits, enable students to have opportunities to take part in ‘hands-on’ and more ‘self-directed’ learning.

A qualitative study with 38 secondary science teachers and four NHMs across England explored the role of NHMs as they collaborated with schools to support school science learning for 11–18 year olds (Collins & Lee, 2006). Data were collected from student surveys and focus groups at the museums and from teacher interviews. Findings showed that enabling access to resources not available to schools had the potential to inspire students by making them more inquisitive about the natural world. Creating opportunities for students to engage with scientists was found to be useful in supporting the new Key Stage 4 curriculum changes (for 14–16 year-olds) as well as being important for informing young people about career choices. There were a number of curriculum areas which teachers identified that would especially benefit from resources and expertise available from the NHMs, including evolution, earth science, classification/taxonomy, and the broader concepts of ‘ideas and evidence’ and ‘how science works’.

## NHMs: new approaches to dealing with challenging topics

Over the last few decades, museums have increasingly sought to be relevant and to benefit society in ways beyond their traditional collecting, preserving and educating activities (Silverman, 2010). Museums are developing educational programmes and exhibits that aim to change visitor knowledge, attitudes and behaviours. In this section, we review the research literature on particular life science topics, exhibits and projects undertaken at NHMs, those using physical artefacts as well as digital technologies. We pay especial attention to topics that some consider to be controversial, such as evolution and climate change.

Evolution is an example of a difficult concept that many NHMs try to convey to their visitors. There is increasing evidence to suggest that an effective way of increasing learning about evolution is exhibitions that have multiple components (Spiegel et al., 2012; Tare, French, Frazier, Diamond, & Evans, 2011), particularly if they are rooted within meaningful narrative (Evans, 2013). Iconic exhibits of impressive fossilised specimens attract hundreds of thousands of visitors to NHMs across the world (Asma, 2001) and these awe-inspiring authentic objects invite the public to experience the story of nature (Conn, 1998). Fossils derive their power from their authenticity, being ‘invested with knowledge’ (Conn, p. 9). However, unlike human-made artefacts, objects of nature do not easily speak for themselves (Evans, Mull, & Poling, 2002). Their meaning is revealed by the interpretive context in which they are placed, usually reflecting the perspective of the curator. At the same time, visitors also bring their own interpretive stance (Friedman, 2005). Successful exhibits integrate these dual perspectives and provide guide notes that inform, ask questions and share uncertainties about the exhibits (Gurian, 1999; Roberts, 1997).

In a survey of a representative sample of almost 400 visitors to US museums, Storksdieck and Stein (2006) found that in comparison to the general public, museum visitors were slightly more likely to endorse an accurate definition of evolution (56% of visitors vs. 48% among the general public) and to report familiarity with the topic (90 vs. 83%). When asked whether evolution was an accurate account of human origins, museum visitors were substantially more likely to endorse this statement (49 vs. 27%) and to agree that science museums should present exhibits on evolution (59 vs. 27%). In a comparative study of museum visitors in Australia, Britain, Canada and the US, Abraham-Silver and Kisiel (2008) found that there was widespread confusion regarding the mechanisms of evolution, in particular natural selection, with 75% of visitors misunderstanding the topic; these misunderstandings were unrelated to the educational level of the visitors or to their acceptance of evolutionary origins (see also MacFadden et al., 2007; Spiegel, Evans, Gram, & Diamond, 2006). Even biology teachers or students who have taken relevant courses and who might be expected to have a firm grasp of the topic have difficulty understanding evolutionary mechanisms as well as difficulty accepting the idea. For example, Nehm, Kim, and Sheppard (2010) compared matched samples of US biology and non-biology teachers and found comparably high levels of misunderstanding of natural selection. These kinds of misunderstandings have been found in diverse student populations from graduate (Gregory & Ellis, 2009) to medical students (Bishop & Anderson, 1990) to child dinosaur experts (Evans, 2000) and school students (Oliver, 2011). In addition, Nehm and his colleagues reported that about 50% of both US biology and non-biology teachers endorsed the inclusion of creationist ideas (‘God created all species individually’) in school curricula.

The theory of evolution is challenging to understand (quite apart from alternative viewpoints that are advanced by certain religious groups), and neither the formal nor the informal educational communities have been particularly successful at conveying these ideas. As the curators and caretakers of the evidence for evolution, it may fall to NHMs to present this evidence in a way that acknowledges the interpretive difficulties of the average visitor and the particular challenges for those who reject evolution (Reiss, 2017). This is particularly the case for palaeontological presentations that emphasise anatomical relationships. While there have been few formal research studies documenting the success of a palaeontological approach, Diamond and Scotchmoor (2006) found that the main foci of major evolution exhibits in museums around the world aligned with the prevailing US national science education standards of that period, of geological time, fossil assemblages, systematics, evolutionary mechanisms and an historical approach. Some of the larger museums in the US, UK, France, Australia and Russia have devoted entire galleries to evolutionary mechanisms, incorporating them into broader evolutionary themes (Diamond & Scotchmoor, 2006). This means that the evidential basis for these presentations has shifted from the more iconic and easily visible fossils to the less easily visualised molecular evidence, in particular DNA. Although an understanding of genetic evidence is part of the science standards for older students, it is not easily grasped by a younger audience and exhibits highlighting such evidence are less likely to captivate visitors.

‘Life through time’ exhibits*,* such as *Evolving Planet* at The Field Museum in Chicago, offer visitors a clear pathway through geologic time (‘… awe-inspiring journey through 4 billion years of life on Earth’ http://www.fieldmuseum.org/), exposure to extinct species and some sense of the relationships between species change and environmental change. Even though most children and adults have great difficulty in understanding the concept of geological time (MacFadden et al., 2007), such time-based exhibits do provide an easy-to-follow linear narrative that allows visitors to encounter macroevolutionary change against the backdrop of an ever-changing planet.

If such exhibits display the geological succession of single exemplars of extinct fossils from one taxon (horses, for example) they can, however, elicit teleological beliefs, whereby individual adaptive changes are thought to lead to progressive changes in successive generations. Assemblages of fossils in outdoor sites, such as the Dinosaur National Monument in Colorado (Diamond & Scotchmoor, 2006), provide an opportunity to observe fossils *in situ* and observe natural variation in a population, which can help counter the idea of ‘progression’ in a species and of individuals changing over time. Moreover, such sites often provide an opportunity to see the scientists at work. This approach is thus more likely to engage younger visitors interested in fossils and the process of fossilisation, which, in contrast to dinosaur expertise, is a positive predictor of evolution understanding (Evans, 2000). Although these sites are often in remote areas, increasing numbers of NHMs now deliver such an experience in-house, by providing visitors with opportunities to observe palaeontological laboratory work in progress (e.g. the Carnegie Museum of Natural History), or even to participate in fossil ‘digs’. One of the more popular exhibits embracing the historical approach was mounted by the American Museum of Natural History on ‘Darwin’ (Diamond & Scotchmoor, 2006). This particular exhibit portrayed Darwin’s life work, including his study, his writings and his struggles as he began to articulate his theories. Notably, the exhibit included a series of short videos with contemporary scholars describing the scientific approach, and how religion and science can be reconciled, thus addressing a widely-held ‘controversial’ view of evolution.

The curatorial organisation of the evidence for evolution according to evolutionary theories of biological diversity and evolutionary relationships (systematics) may make more sense to the budding biologist than to other visitors. If, like the American Museum of Natural History (Diamond & Scotchmoor, 2006), museums that take this approach have an abundance of fossils illustrating the entire fossil record, then this approach has the virtue of avoiding the presentation of a single fossilised representative of a particular species, which is as likely to mislead as inform. Museums that have access to fossil hominin specimens (and even those that do not) can mount informative exhibits on human evolution (Scott, 2005). Despite the attraction of such exhibits, the late appearance of modern humans in the fossil record can give the impression that evolution is directional and progressive with *Homo sapiens* as the endpoint, especially to visitors with preconceived beliefs. Moreover, the relationship between humans and other primates is particularly problematic for visitors with strong creationist beliefs (Tare et al., 2011). Thus, merely including human evolution in exhibits is not sufficient; it is necessary to carefully organise the evidence and demonstrate that *Homo sapiens* is one species among many (Scott & Giusti, 2006). In *Evolving Planet* (The Field Museum in Chicago), the hominin story is intentionally placed at the midpoint of the final gallery – rather than at its end – for this very reason (R. Kissel, personal communication).

Members of the public are more likely to concentrate on mammal, particularly primate, exhibits and hurry past invertebrate collections, as shown by an early 1938 time and motion study at the Peabody Museum (Logan & Pickering, 2014), thus evading the intent of the curatorial staff and incompletely experiencing the whole story of evolution. What interests curators may not exert the same appeal for visitors.

*Explore Evolution* (Nebraska Museum of Natural History and six other US Midwest museums) provided a contemporary touch, using as an organisational narrative the ongoing research of the scientists investigating the evolution of a variety of organisms from a virus to a whale (Diamond, Evans, & Spiegel, 2012). This approach bridged the gap between the more customary palaeontological exhibits and those focusing on the molecular level by presenting both the genetic and fossil evidence for each organism. Pre-post studies of the visitor experience indicated that this approach successfully conveyed basic evolutionary science concepts (Spiegel et al., 2012). *Charlie & Kiwi’s Evolutionary Adventure* (New York Hall of Science) used a story about ‘Charlie’ to focus on the evolution of birds from dinosaurs. The exhibition was designed for 7–10 year olds, travelled to a variety of informal institutions and included a children’s book. Children who visited the exhibition were more likely than their peers who visited a different exhibition to grasp the basics of natural selection (Evans, Weiss, Lane, & Palmquist, 2016). The *Great Debate* Programme at the Natural History Museum in London was designed to involve students in the *1860 Oxford Evolution* debate; students adopted the roles of the main participants in the debate and on a field trip to the museum they were introduced to the evidence by trained facilitators. In comparison with their peers, students who attended the museum programme increased their understanding of evolutionary concepts (Tenenbaum, To, Wormald, & Pegram, 2015).

Exhibitions that focus on health issue*s* from an evolutionary perspective have the goal of tying evolution to visitors’ everyday interests. For example, the Yale Peabody Museum developed programmes on Lyme disease (endemic in that area) and West Nile disease (Pickering, Fawcett, & Munstermann, 2012), and proved popular with visiting students. Pre-post evaluation of student understanding indicated that the exhibit/programmes were successful in conveying evolutionary concepts to the majority of students. Similarly, an exhibit titled *Evolution Health Connection* took as its main focus the science of evolutionary medicine in which the exhibit portrayed the ongoing evolutionary processes in human populations and the influence of evolutionary principles on human health and disease. Adult and teen visitors enjoyed the experience and, in comparison with their peers, who visited a control exhibit, they were much more likely to grasp the idea that evolutionary processes contributed to modern health problems (Weiss, Evans, & Palmquist, 2016).

Visitor experiences in NHMs and other informal science institutions are impacted by both open-ended exploration play and interactive exhibits (Allen, 2004; Allen & Gutwill, 2004; Crowley et al., 2001; Humphrey & Gutwill, 2005; Oppenheimer, 1976). Interactive exhibits usually allow visitors an opportunity to explore and/or manipulate physical artefacts, phenomena or specimens. Digital media have increasingly become popular as a way of enhancing visitor experiences by creating new types of hands-on experiences (e.g. via the use of videos, photos, texts, puzzles and games). Recent moves to offer visitors opportunities to explore visualisations of large scientific datasets have been welcomed (e.g. Block et al., 2012; Louw & Crowley, 2013; Ma, Liao, Ma, & Frazier, 2012; Roberts, Lyons, Cafaro, & Eydt, 2014). Exhibits that offer these types of manipulation have several advantages, enabling hands-on experiences that use digital and computational tools and allowing visitors to learn scientific concepts (Louw & Crowley, 2013; Ma et al., 2012).

Video films have been used by NHMs to help educate visitors about evolution (e.g. Prum, 2008), and these and non-interactive exhibits such as evolutionary (or phylogenetic) tree diagrams used to communicate the relatedness of organisms and the evolution of traits and structures over time are reported to be difficult to understand (MacDonald & Wiley, 2012; Meir, Perry, Herron, & Kingsolver, 2007; Novick & Catley, 2012; Phillips, Novick, Catley, & Funk, 2012). This is particularly the case when concepts presented by the phylogenetic tree diagrams are in conflict with people’s own beliefs about evolution (Novick, Catley, & Funk, 2011). An effective way to help visitors learn is thought to be the inclusion of exhibits that enable social and physical engagement (Crowley et al., 2001; Eberbach & Crowley, 2005; Falk & Dierking, 2000). For example, researchers exploring dyad interactions elicited by interactive tabletop exhibits learn more about how social and physical engagement shapes visitors’ learning at computer-based exhibits (e.g. Davis et al., 2015; Horn et al., 2016).

**Figure 1.**
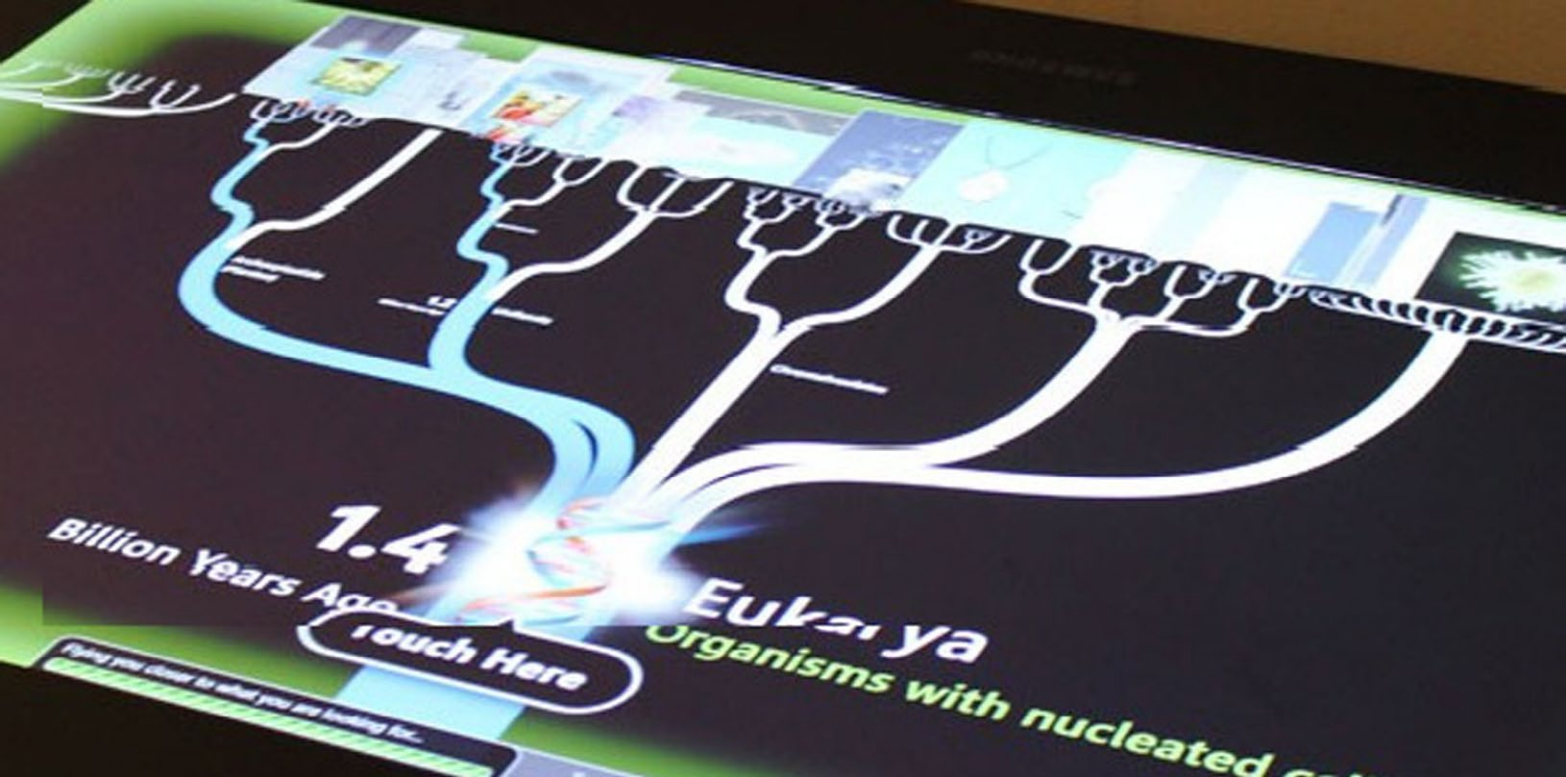
DeepTree phylogenetic tree of life.

A recent exhibit, *DeepTree,* involves the visualisation of large scientific datasets by integrating phylogenetic and species data from five publicly available data sources. This allows visitors to explore a phylogenetic tree of life that contained over 70,000 species. With a deep zoom interaction technique, users are able to learn about the origin and unity of life, and the diversity of species on the planet. Whilst exploring *DeepTree,* users encounter a range of evolutionary landmarks such as the emergence of nucleated cells and of jaws (Figure 1).

In a study of the effectiveness of the *DeepTree* exhibit at two NHMs, researchers recruited 248, eight to 15 year-olds, in dyads, who were randomly assigned to one of four conditions. In the first two conditions, the dyads interacted with different versions of *DeepTree* for ten minutes; in the third condition, dyads watched a ten-minute video on the same evolutionary topics. The final condition was a baseline control. The dyads were video-recorded so that researchers could collect measures of oral engagement, which were used in conjunction with computer logs of touch interaction collected in the interactive exhibit. Follow-up interviews assessed understanding of both macro- and micro-level evolutionary concepts. In comparison with the baseline condition, dyads in both of the *DeepTree* conditions were significantly more likely to endorse the idea that all species on Earth were related through common ancestry, use evolutionary terms and concepts in their explanations, and correctly interpret a phylogenetic tree diagram. Moreover, the learning conditions that seemed optimal occurred when the dyads both activated the relevant interactive exhibit functions and conversed about the specific experience. Statistical analyses controlled for participants’ ages, family backgrounds and prior knowledge. These results provide strong evidence that interactive tabletop exhibits can provide effective learning experiences, even if the interaction is relatively brief.

This sort of research suggests that interactive environments can help enhance museum experiences and learning, and confirms the importance of visitor social interaction which underpins enhanced learning in free-choice environments (Ash, 2004; Crowley et al., 2001; Eberbach & Crowley, 2005; Falk & Dierking, 2000; Falk & Storksdieck, 2005). Multi-touch tabletops like *DeepTree* are increasingly being seen as a particularly effective way of helping people learn about all forms of science as young learners’ attention switches between exploring technical aspects of the system and learning concepts, whilst also being able to entertain themselves (Price & Pontual Falcão, 2011). It has been argued that the process around shared interfaces between learners enhances collective knowledge construction and argumentation and that interference leads to students changing their course of action and/or integrating the choices of others and/or ignoring/undoing the actions of others (Davis et al., 2015; Price & Pontual Falcão, 2011).

The design of *DeepTree* was influenced by similar digital multi-touch displays that focus on helping visitors learn about biological concepts and evolution. The two learning environments *Phylo*-*Genie* (Schneider et al., 2012) and *G*-*nome Sur*fer (Shaer et al., 2011) introduce students to evolution, genomics and tree-thinking (Baum, Smith, & Donovan, 2005), with the use of a combination of tangible and multi-touch tabletop technologies. *Build*-*a*-*Tree* is another example of a phylogenetic tree-thinking game based on multi-touch tabletop technology in an NHM (Horn et al., 2012). Visitor interaction with *Build*-*a*-*Tree* showed that the use of social interaction through game play contributed to visitors reporting that they had had an engaging and enjoyable learning experience. Similar findings have been found with a range of digital tabletop interaction displays, e.g. *Futura*, a tabletop game on issues of environmental sustainability (Antle, Tanenbaum, Seaborn, Bevans, & Wang, 2011).

Many NHMs undertake significant work on climate change (Cameron, 2011). Alongside other informal science institutions, such as zoos, aquaria and science centres, NHMs now see environmental issues, climate change and sustainable development as essential mission-related topics, where collections are an irreplaceable resource to support such research and public communication. Responding to climate change and other environmental issues has become a common topic for discussion within the profession, and specialist groups have been established, such as the Museums and Climate Change Network, launched in 2013, (http://mccnetwork.org). The public understanding of and attitudes towards climate change informs museum practice in this area. In 2008, the Association of Science-Technology Centres (which includes many NHMs as members) partnered with the *Yale Project on Climate Change Communication* to survey museum visitors to understand what they knew about the climate system and the causes, impacts and potential solutions to global warming (Yale Project on Climate Change Communication, 2011). Perhaps not surprisingly, frequent visitors to informal science institutions have a better understanding of these issues than non-visitors, are more likely to consider climate change as real and are supportive of personal and political actions to mitigate the threat. However, even within this segment of the public, relatively few had an in-depth understanding of climate change. The study did find that respondents think informal science institutions are trusted sources of information, and that the majority of visitors are interested in learning more about climate change. An extensive survey (Cameron, 2012) was conducted in Australia and the US to probe the roles of NHMs and other types of informal science institutions in public understanding of these issues. It revealed that the public believes museums are places to obtain impartial information and found that many visitors thought museums should be actively engaged in public discourse around these topics, particularly in facilitating discussion and being part of networks of organisations concerned with climate change.

Much of the literature on NHMs and climate change centres on what museums are (or should) be doing in this arena. For example, a special issue of the *Journal of Museum Education* (Anderson & Williams, 2013) examined the best ways that museums can empower visitors to create a positive future, and included several papers on climate change. A special issue of *Museum and Society* (2011) looked at how museums can communicate and foster an understanding of climate science. Museum research to explore the effectiveness of individual climate change and environmental programmes may be conducted for specific programme improvement rather than generalisable knowledge. Examples include the summative evaluation of the American Museum of Natural History’s major travelling exhibit *Climate Change* (People, Places & Design Research, 2009) and the summative evaluation of *POLAR*-*PALOOZA*, a programme implemented across the US (Selinda Research Associates, 2010). As in much of the work in assessing the impact of informal science programmes, such studies are focused on short-term affective and cognitive outcomes rather than long-term impacts. The National Network for Ocean and Climate Change Interpretation (NNOCCI) is a collaboration of ISL institutions and climate and learning science researchers; it aims to train front-line ISL institution educators and volunteers to communicate about climate change using techniques informed by research in the cognitive and social sciences (Spitzer, 2014).

While much of the work of NHMs takes place *in situ*, the creation of online catalogues, such as cybercabinets, has brought a new dimension to the exhibiting of artefacts, outreach activities and educational programmes. Digital media are providing museum educators and exhibit developers with a new suite of tools for accomplishing their communication goals (e.g. Loveland, Buckley, & Quellmalz, 2015). Indeed, teachers and their students are increasingly relying on distance materials produced by NHMs and other ISL institutions. NHMs have an increasing presence on the web and are aware that in order for their information to be valuable, the public needs to be able to find, access and use their resources (e.g. Woods, 2007). The development of cybercabinets that incorporate eight key principles of utility has been recommended to NHM curators: be useful; be beautiful; keep it personal; provide serendipity; share; encourage participation; provide access to experts; and collaborate (Sargent, 2014). Enabling online users to have access to experts may encourage users to return to online museum resources (Howes, 2007; MacArthur, 2007). Even multi-institutional collaborations are created when cybercabinets capitalise on online networking (Weinberger, 2012).

## Opportunities for the professional development for teachers

A feature of some ISL institutions, including NHMs, is their hosting of professional development programmes for teachers to help them make the most effective use of their time when their students visit. Such programmes create opportunities for teachers to engage in content learning, to acquire different pedagogical strategies and to learn more about available resources. The Informal Learning Collaborative (ILC) programme, a professional development programme for informal educators, is currently leading teacher development programmes (Bevan et al., 2010) with the aim of building a community of informal educators. These educators are working closely with schools, with the resources of ISL institutions, and with design experts in professional development programmes. One programme based at the Exploratorium in San Francisco has worked with over 100 informal educators who represent around 60 institutions and communities in the US and the UK.

Teachers and schools value visits to museums as they present opportunities for students to learn about scientific concepts and take part in science in ways that are not possible in schools. Despite teachers’ positive views about NHMs, more can be done to help them get the most out of their interactions with them. Even in the early 1980s, there was a recognition by the academic community that in order to make museum trips successful, teachers need to be able to organise, sequence, focus and evaluate the event according to the needs of their students, while also ensuring that the trip itself leads to particular planned learning goals (Muse, Chiarelott, & Davidman, 1982). The evidence shows that some teachers do not plan their visit, nor do they define learning goals for the visits or view museum activities as a sociocultural learning experience (Cox-Petersen et al., 2003; Griffin, 2004; Griffin & Symington, 1997; Kisiel, 2003; Price & Hein, 1991).

Where teachers engage in meaningful activities that focus on museum learning, encouraging results emerge from the collaborative efforts (Anderson, Lucas, Ginns, & Dierking, 2000; Gilbert & Priest, 1997; Henriksen & Jorde, 2001). ISL institutions can play an important part in science teachers’ professional development. One US survey study found that a large proportion of ISL institutions played a key role in the professional development of K-12 teachers with nearly 60% of all informal science institutions and 81% of science centres providing science professional development (Center for Informal Learning & Schools [CILS], 2005). Furthermore, the professional development that teachers received from ISL institutions was of greater value and quality than the professional development they received from other sources (Dorph, Shields, Tiffany-Morales, Hartry, & McCaffrey, 2011; Dorph et al., 2007). Collectively, the scale of this work is quite significant (CILS, 2005). In the US, as in some other countries, ISL institutions have been involved in developing school curriculum materials for many years, and some of these institutions provide online materials to support teachers. There are a number of reasons why teachers do not spend time planning for museum visits. Griffin (2004) reports that logistical issues, time constrains, various student needs and pressure for accountability are the key professional factors that limit teachers’ ability and willingness to provide proper preparation and post-visit activities. NHMs promote, deliver and support teachers through courses and workshops led by NHM educators and scientists (e.g. https://hmnh.harvard.edu/resources, https://nhmu.utah.edu/educators/workshops); with such courses and workshops are recognised and accredited as appropriate professional development opportunities for school teachers.

## Recommendations

This review of student learning through NHMs has shown that they can make an important contribution to students’ learning and engagement in science. Of particular note is their increasing ability to integrate digital resources with natural artefacts and to engage students with major issues in contemporary science, such as evolution and climate change, that are frequently contentious. We focus on three main and interrelated recommendations: NHM exhibits and other learning provision; professional development of teachers and their use of NHM resources; and the need for appropriate measures that capture the impact of NHMs.

Museum experiences can help to develop students’ scientific skills and understanding of science whilst also helping them to develop an enquiring and critical attitude towards science, to engage with it and to consider the possibility of a career in it. Curated artefacts, interactive displays and digital technologies all feature in NHMs. Many offer opportunities to showcase scientists actively engaged in doing science’. Key ways in which technology could further enhance the visitor experience is by enabling visitors to get closer to the artefacts showcased in museums and to help facilitate shared experiences and knowledge growth (Hanko, Lee, & Okeke, 2014). In addition, we see great potential for NHMs to make more of their collections available on-line for students to study; for example, students could examine intraspecific variation within populations, across geographical areas and over times.

Given the extensive research indicating that cultural, educational and cognitive factors influence students’ understanding of science, more attention should be given to students’ prior knowledge and resulting interpretive stances in the design of exhibits and related learning experiences. When developing programmes, there needs to be a focus on the cognitive and learning literature to understand and help develop learning goals and learning progressions. Likewise, ensuring an appropriate ‘connect’ with learning that feeds on students’ intrinsic motivation would be valuable in increasing students’ science learning. For students to learn effectively when on museum visits they should have the opportunity to explore exhibits interactively with others as well as on their own (cf. Andre, Durksen, & Volman, 2017) and the opportunity to discuss what they are learning with their peers, museum educators and their teachers. Importantly, they should also be encouraged to make links with knowledge they already have about scientific issues. In order to do this well, students need to spend some time understanding the focus of the learning goals both before and after the visit.

A committee of experts from STEM organisations in the US investigated how both ISL and school institutions could improve young peoples’ learning in STEM, recommending how strategic connections among the diverse communities could help establish new avenues of teacher preparation and professional development, more comprehensive assessment of knowledge, skills and attitudes about STEM and contribute to the development of a more integrated curriculum (National Research Council, 2014). One possible way of achieving enriching NHM visits is for teachers themselves to receive guidance in what they should be doing in such settings (Griffin, 2004) and develop their own knowledge of the science behind the exhibits through interaction with museum scientists as part of their own professional development.

There is little in the extant literature that adequately describes, measures or captures the long-term impact of NHMs on the learning of students. While it is relatively straightforward to record changes in knowledge using pre-post assessments either side of NHM visits and to gather feedback on the ‘experience’, it is less easy to measure any long-lasting changes to affect or understanding that have been elicited by the immersion in a whole topic exhibition. There needs to be a greater focus on such long-term impacts and, in addition, how such impacts differ from short-term ones. Bell et al. (2009) also suggested that students’ learning outcomes ought to focus not only on the way an individual learns but on how whole groups learn collectively. Likewise, students’ backgrounds, interests and motivations to learn science are known to vary but how these are impacted by NHM experiences is much less understood. Clearly, more research needs to address how an NHM intervention (visit, use of resources etc.) is used, planned for and evaluated.

## Conclusions

This review reveals how NHMs can support and be part of teachers’ and students’ learning and engagement in science. NHMs can support and be part of teachers’ and students’ learning and engagement in science both by exhibiting artefacts and using digital technologies. NHMs can provide students with new knowledge and perspectives in well-designed exhibits, with impacts that can last years. One effective way of using NHM resources to enrich science teaching and learning is for NHMs to collaborate with the formal sector. The most fruitful way of ensuring change is by implementing long-term programmes, which offer learning experiences that schools are not able to provide. Successful programmes with clear evaluations (cf. Fu, Peterson, Kannan, Shavelson, & Kurplus, 2015) capture the richness of the NHM experience with both cognitive and affective measures, in part because students’ intrinsic motivation (e.g. expressions of interest, enjoyment, enthusiasm) is likely to be strongly related to science engagement in the classroom. With collaborations between teachers and museum scientists and educators, to better understand students’ prior learning experiences, it is likely that students will benefit from focused tasks *in situ* within a structured and age-appropriate curriculum that is largely school-based: a visit needs to ‘fit’ well within the planned curriculum. While digital technologies enable museum materials to be presented and accessed off site, museum visits housing local and national collections provide a unique experience for school students to engage with large exhibits that illustrate the macro- and micro-aspects of a topic.

## Notes on contributors

***Tamjid Mujtaba*** is a Senior Research Officer at UCL Institute of Education, University College London and Associate Editor of *London Review of Education*. She has worked on a range of research projects which cross psychology and education; presently she is a co-directing a five year research evaluation which aims to increase the number of students continuing with chemistry post-16.

***Martin Lawrence*** was until recenlty Head of Learning Strategy and Research at the Natural History Museum, London. A former inner London primary school teacher with an academic background in natural sciences and science communication, he has spent the last twenty years developing and managing education teams and learning activities at the Museum.

***Mary Oliver*** is an experienced science teacher and educator, having worked in England, Denmark and Australia. She works at the University of Nottingham with pre-service teachers and researches students’ learning as Associate Professor in Science Education. She is vice-chairman of the International Biology Olympiad, promoting the study of Biology across the world.

***Michael J. Reiss*** is Professor of Science Education at UCL Institute of Education, University College London and a Fellow of the Academy of Social Sciences. The former Director of Education at the Royal Society, he has written extensively about curricula, pedagogy and assessment in science education.

## Disclosure statement

No potential conflict of interest was reported by the authors.

## Funding

Funding was provided by the Wellcome Trust as part of the Science Learning+ initiative [grant number WT106015MA].
